# PeptideMine - A webserver for the design of peptides for protein-peptide binding studies derived from protein-protein interactomes

**DOI:** 10.1186/1471-2105-11-473

**Published:** 2010-09-22

**Authors:** Khader Shameer, Lalima L Madan, Shivamurthy Veeranna, Balasubramanian Gopal, Ramanathan Sowdhamini

**Affiliations:** 1 National Centre for Biological Sciences (TIFR), GKVK Campus, Bellary Road, Bangalore, 560065, India; 2 Molecular Biophysics Unit, Indian Institute of Science, Bangalore 560 012, India

## Abstract

**Background:**

Signal transduction events often involve transient, yet specific, interactions between structurally conserved protein domains and polypeptide sequences in target proteins. The identification and validation of these associating domains is crucial to understand signal transduction pathways that modulate different cellular or developmental processes. Bioinformatics strategies to extract and integrate information from diverse sources have been shown to facilitate the experimental design to understand complex biological events. These methods, primarily based on information from high-throughput experiments, have also led to the identification of new connections thus providing hypothetical models for cellular events. Such models, in turn, provide a framework for directing experimental efforts for validating the predicted molecular rationale for complex cellular processes. In this context, it is envisaged that the rational design of peptides for protein-peptide binding studies could substantially facilitate the experimental strategies to evaluate a predicted interaction. This rational design procedure involves the integration of protein-protein interaction data, gene ontology, physico-chemical calculations, domain-domain interaction data and information on functional sites or critical residues.

**Results:**

Here we describe an integrated approach called "PeptideMine" for the identification of peptides based on specific functional patterns present in the sequence of an interacting protein. This approach based on sequence searches in the interacting sequence space has been developed into a webserver, which can be used for the identification and analysis of peptides, peptide homologues or functional patterns from the interacting sequence space of a protein. To further facilitate experimental validation, the PeptideMine webserver also provides a list of physico-chemical parameters corresponding to the peptide to determine the feasibility of using the peptide for *in vitro *biochemical or biophysical studies.

**Conclusions:**

The strategy described here involves the integration of data and tools to identify potential interacting partners for a protein and design criteria for peptides based on desired biochemical properties. Alongside the search for interacting protein sequences using three different search programs, the server also provides the biochemical characteristics of candidate peptides to prune peptide sequences based on features that are most suited for a given experiment. The PeptideMine server is available at the URL: http://caps.ncbs.res.in/peptidemine

## Background

Integrated approaches in bioinformatics have become an important step in the process of knowledge discovery in life science. Thus, bioinformatics is now in a transition stage, from a data-centric component of biology to a knowledge-based science. This transition is consistent with a more pro-active role for bioinformatics approaches in supporting experimental strategies based on *a priori *information. The complexity of biological systems requires efficient integration of data, tools and protocols to extract new information. While high-throughput data integration approaches in bioinformatics have provided new insights into several biological problems, these are often not in a form suited for specific experimental validation. These data nonetheless include additional levels of function associations for proteins and their orthologs alongside enhanced annotation of gene products to aid in the functional characterization of proteins [1-6]. Bioinformatics tools that utilize this information and provide well-defined, experimentally verifiable data are clearly needed to translate these *in silico *predictions into a validated set of functionally annotated multi-protein interactions. Data integration approaches can provide new avenues to understand molecular interactions and aid the design of new experiments to identify interesting molecular players. Large-scale data integration, data mining and semantic approaches in bioinformatics could accelerate such endeavours [7-13].

We report a new method and an associated web server for designing peptides by utilising protein-protein interaction data from the perspective of '*interacting sequence space*'. The rational approach of PeptideMine is based on sequence search in interacting sequence space and integration of an array of data and tools that help in designing the peptides which can be used for experimental or computational studies. Various examples are provided where the PeptideMine server and this approach can be used. Biochemical validation is performed to show that the peptides derived from PeptideMine searches are most suited to drive new experimental studies. From a technological perspective, the PeptideMine server is an ideal example of a bioinformatics mashup. Mashup [14] refers to a web-based application that integrates data or functionality from different external applications to create a new application. The PeptideMine server thus signifies a step forward in the development of a bioinformatics mashup [15] by integrating tools, databases and resources to develop a web-based platform to identify and analyse peptides from interacting partners that are suitable for protein-peptide binding studies.

### Concept of PeptideMine

This paper describes a strategy to utilize protein-protein interaction data, primarily based on protein sequences, to identify putative peptides and functional motifs in potential interacting partners of a given protein. PeptideMine is an integrated and unified resource that sensitively combines sequence searches in the 'interacting sequence space' of a protein using sequence patterns or functional motifs. We define 'interacting sequence space' as the sequences of interacting partners of a given protein obtained from a database of protein-protein interactions. A compilation of indices that describe the chemical and solubility properties of candidate peptides is also provided to facilitate further investigation by *in vitro *or *in silico *studies. Furthermore, the biological significance of such a design-strategy is highlighted in the context of domain-domain interactions and function annotations. This integrated search approach, called "PeptideMine", is completely automated and a webserver is implemented [16] primarily for experimental and computational biologists.

### The PeptideMine server: a web-based platform for the identification and analysis of peptides and functional motifs from interacting proteins

Several bioinformatics methods are currently available for peptide identification based on sequence patterns, biological context, and structure (for a detailed account of available methods, tools and resources: please refer to the reviews [17-19]). Various databases are also available that provide information about different aspects of protein-peptide interactions [20-22]. While most databases and bioinformatics resources employ simple pattern-searching techniques to identify potential interacting partners, the PeptideMine approach differs from these computational methods.

PeptideMine server integrates concept of sequence searches in interacting sequence space with various resources like Gene Ontology (GO) annotations, domain-domain interaction data and a set of different tools to assess various properties of peptides that can further add annotations to the peptides or patterns mined using the approach. Protein sequence patterns can be searched using the PeptideMine server at different levels. These include a standard PeptideMine Search, BLASTP [23] and regular expression or PROSITE [24,25] based pattern search using ScanProsite [26]. The peptides thus identified can be further examined for different features like molecular weight, pI, instability index [27], Grand Average of Hydropathy (GRAVY) [28], charge, amino acid composition and molar absorption coefficient. The user can also generate and analyse amino acid index-based plots for the peptides using 516 amino acid indices reported in the AAindex Database [29,30]. Functional annotations of the interacting proteins are provided using the organism-specific GO annotations [31-33] of the corresponding gene and each peptide is further scanned for potential PROSITE patterns [24,25]. The predicted secondary structural elements (using PSIPRED [34] as well as disordered regions predicted by DISOPRED [35]) are also provided to examine the conformational feasibility of a given interaction. To further screen the peptides as potential candidates for *in vitro *or *in silico *studies, the server also maps the location of the peptide on to its domain. Thus, potential domain-domain interactions are assigned to the peptide-containing domains and its interacting segment in the query protein. The protein-protein interaction data used in PeptideMine is resourced from the STRING database version 7 [4] and the domain-domain interaction data is obtained from DOMINE database [36]. In the PeptideMine server, domain architectures of a query protein and its interacting partners are elucidated using hmmpfam from the HMMER suite [37] using an E-value threshold of 0.01. Following the peptide search and enumeration of various parameters associated with peptides, the server provides domain-domain interaction information based on the protein domain containing the peptide in the query sequence. For example, if a peptide identified using any of the search programs is observed to be a part of a domain encoded in the sequence of an interacting partner (this is achieved by mapping the location of peptide to the domains predicted in the sequence of interacting partner), an hmmpfam search is performed on the query sequence to predict the protein domains in the query sequence. Further, this information is used to search for the probable domain-domain interaction between these domains. Information on the interaction between domains (where one of the domains includes the query sequence pattern) to identify the location of the peptide in the query sequence is obtained from the DOMINE database.

### Search programs in PeptideMine Server

**Figure 1:**
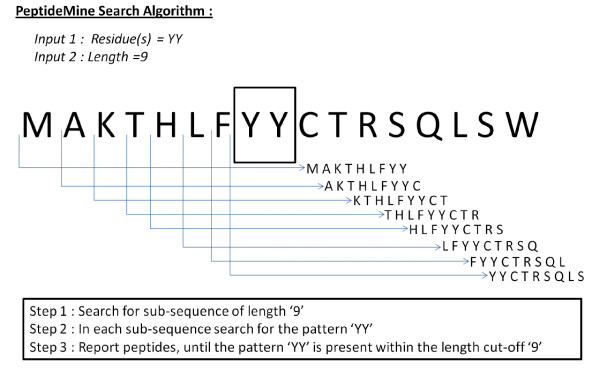
**Graphical representation of the 'PeptideMine search' algorithm**.

**Figure 2:**
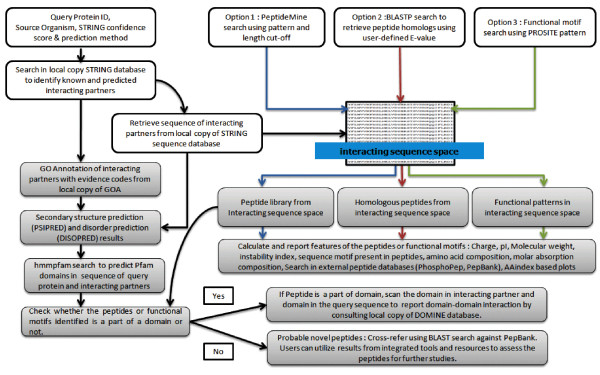
**Detailed flow-chart of the integrated method used in PeptideMine server**.

PeptideMine offers three search options for users to search the interacting sequence space (sequences of interacting partners of query protein) for a given protein. The user can identify peptides from sequences of interacting partners using different sequence search programs including BLASTP and ScanProsite [26]. A detailed description about BLASTP[23] and ScanProsite is available elsewhere [26]. In the first search option, referred to as the 'PeptideMine Search', peptides are searched based on two criteria (a residue or a stretch of residues and the length of the peptide sequence). Using these criteria, the 'PeptideMine Search' program identifies peptides from the interacting sequence space of a query protein based on the input parameters. In a typical search using the PeptideMine search algorithm, the server retrieves interacting partners for a query protein from a local copy of the STRING database using the criteria set by the user. The sequence of the interacting partner(s) is obtained from the database of sequences of proteins reported in STRING. Each protein sequence thus identified is further scanned for the desired parameters (for example residue(s) = YY and length = 9) to identify potential peptides. These searches are performed using a sliding window with a step size of one residue. This approach is important in the selection of peptides as a single residue change (point mutation) could lead to substantial differences in the solution properties of a peptide. A graphical representation of the 'PeptideMine search' algorithm is given in Figure 1. A detailed flow-chart of the method used in PeptideMine server is provided in Figure 2.

### Scope of PeptideMine

The search options in the PeptideMine server would find three major applications. These include (a) Design of a peptide library based on the interacting sequence space of a protein; (b) Search for homologues of known peptides in the interacting sequence space of a protein using a BLASTP search; and (c) Identification of a functional motifs or putative binding sites using ScanProsite in the interacting sequence space based on either PROSITE patterns or user-defined motifs. Peptides identified using "PeptideMine", in general, would find applications in experimental and computational studies to determine binding affinity and/or specificity, peptide models for kinetics measurements using surface plasmon resonance (SPR) or in computational strategies such as protein-peptide docking.

Information on interacting proteins in a cell is essential to understand cellular and developmental processes. However, the extrapolation of data on protein interactions based on *in vitro *experiments to functional roles *in vivo *is often difficult, primarily, due to artifacts such as concentration effects or unnatural protein conformers (due to the presence of large tags for immobilizing a target protein). Indeed, interactome information based on data mining has a high rate of erroneous identification and is often ill-equipped to identify transient and low affinity interactions. These limitations can be overcome, to an extent, by using the data compiled by the PeptideMine server. The search results from various tools can be used as a filter to screen potential interacting partners. This feature is highlighted in the 'results' page where residues in the design peptide are coloured according to the amino acid classification as surface or neutral or buried. This information can be utilised to remove a potential interacting molecule if it is judged that the peptide motif in the molecule would be unavailable or buried for interaction in the context of a folded protein. Information on the secondary structure, disordered regions, physicochemical properties and GO annotation could also be employed to screen predicted interacting partners. Furthermore, it is possible for the user to exclude interacting partners located in different cellular compartments or expressed in different functional contexts. Since it is impossible for the experimental validation and assessment of a large number of peptides included or removed by these filtering strategies, bioinformatics approaches for the identification and analysis of relevant peptides are of crucial importance. The user can then assess the suitability of identified peptides for a given experiment using standard physico-chemical properties of the peptides and select a subset from the list given by the PeptideMine server. Given the need to balance the priority between examining a biologically relevant interaction and one that is most feasible to examine experimentally, the interacting partner protein is highlighted using Gene Ontology (GO) annotations [31,32]. The PeptideMine approach also permits the identification and assessment of peptides from interacting partners of proteins reported in the STRING database. The current version of PeptideMine server is available for search in six model organisms (*Homo sapiens*, *Mus musculus*, *Caenorhabiditis elegans*, *Drosophila melanogaster*, *Saccharomyces cerevisiae *and *Arabidopsis thaliana*).

## Implementation

### PeptideMine server: description and features

The PeptideMine server is based on the concept of identifying potential peptides for protein-peptide binding studies from a compilation of known and predicted interacting partners of a query protein as reported in the STRING database [4]. The PeptideMine server can be used to search in the interacting sequence space of a given protein using three approaches. It is thus possible for a user to generate a library of peptides from the interacting sequence space of a protein or use the PeptideMine server to search for homologues of known peptides in the interacting sequence space of a protein. Alternatively, the server can be used to search for functional patterns or motifs within the interacting sequence space using PROSITE patterns. The design of this server was prompted by the observation that information on protein-peptide interactions are severely limited by the small number of experimentally validated peptides reported in the literature. A likely cause for this is that the identification of new peptides for a large-scale protein-peptide experimental study is both time-consuming and expensive. The PeptideMine server can be used to identify peptides for both specific as well as high-throughput studies. For a given query sequence or protein ID, the PeptideMine server uses the local version of the STRING database [4] to identify potential interacting partners. The STRING database[4] extracts protein-protein interaction events from different sources such as experimental data, literature, co-occurrence or curated databases and offers a comprehensive set of potential protein-protein interactions that can be tested experimentally; however, the scores could substantially depend upon the method of association and two proteins or domains reported as 'interacting' in the database need not necessarily be engaged in direct physical interaction. Nevertheless, such interactions are interesting candidates for further protein-peptide studies to determine putative binding partners. Using PeptideMine, the user can scan through the sequence of interacting partners and identify new peptides that can be used for protein-peptide studies. Using integrated GO annotation data [31,32] from the EBI-GOA database [33] for six organisms and ontology data from the OBO foundry [38], PeptideMine provides an additional filter that it can help the user restrict a peptide search exclusively from the same class of GO annotations such as a particular biological process or molecular function or cellular compartmentalisation of the protein. PeptideMine also provides a seamless link to the AAindex database [29] via SEQPLOT [30] to evaluate the peptides using 516 amino acid indices. SEQPLOT [30] integrated in PeptideMine, provides an option to generate three different plots with multiple indices in a single page, so that a user can combine different features of peptides like hydrophobicity, hydrophilicity and amphiphilicity in a single window. Domain architecture of the query protein and the interacting partners are obtained using a hmmpfam search against the Pfam Database [39,40]. Pre-computed domain-domain interaction from the DOMINE database [36] is used for the mapping of domain-level interaction based on the occurrence of peptides inside a predicted domain. DOMINE is a database of known and predicted protein domain (domain-domain) interactions inferred from structural entries, and interactions predicted by eight different computational approaches using Pfam domain definitions [36]. Integration of domain architecture and domain-domain interaction of query protein and its interacting partner will be useful to select the peptides for further studies. For example, if the location of a peptide identified using PeptideMine is found to be a part of a well-defined domain, the server uses the domain architecture derived from hmmpfam and then integrates the information from DOMINE database to search if the query protein contains any particular domain that is known or predicted to interact with the domain where the peptide is identified. This option also provides further assurance that a given peptide would be suited for protein-peptide binding studies.

### Database and tool integrated in Peptide Mine Server

PeptideMine is developed using data integrated from five databases, four tools and scripts developed using different bioinformatics software libraries (Bio-* libraries) [41]. This information, including a short description of databases, tools and their applications, is compiled in Table 1.

**Table 1:** Brief descriptions of databases and tools integrated in the PeptideMine server

No	Database/Tool	Description of resource	Application in PeptideMine server
1.	STRING (version 7) [4]	Database of known and predicted protein-protein interaction.	Protein-Protein Interaction data and sequence of interacting partners are sourced from STRING database

2.	Gene Ontology Annotation (for 6 genomes) and Ontology file (.obo) for Biological Process, Molecular Function and Cellular Compartment [33,38]	GOA project provides high-quality GO annotations to proteins reported in Uniprot	GOA files are used to obtain the individual GO annotations of gene products. .obo files are used to obtain description of GO terms based on GO Ids obtained from GOA files.

3.	Pfam (version 22) [39]	Pfam is a database of protein families, represented as multiple sequence alignments and HMM models	Pfam is used as the target database to obtain the domain architecture of query sequence and its interacting partners using hmmpfam.

4.	AAindex (version 7.0) [29]	AAindex is a database of amino acid physicochemical properties, substitution matrices and statistical protein contact potentials.	Data from AAindex is used to generate plots using 516 Amino Acid Indices. An amino acid index is a set of 20 numerical values representing various physico-chemical and biochemical properties of amino acids.

5.	DOMINE (version 1.1) [36]	DOMINE is a database of known and predicted protein domain (domain-domain) interactions. It contains domain-domain interactions reported in PDB along with interactions predicted using various computational approaches using Pfam domain definitions.	Pre-computed domain-domain interaction from DOMINE is used to provide additional support for a protein-peptide interaction. Domine is used in PeptideMine as it reports domain-domain based on the Pfam definitions.

6.	BLASTP (version 2.2.17) [23]	BLASTP or Protein BLAST is a tool for searching protein sequence databases using a protein sequence as query.	BLASTP is used as one of the search programs in PeptideMine server. BLASTP is used to search the database of interacting partners' sequence to obtain homologues sequence in the interacting sequence space

7.	hmmpfam (HMMER suite version 2.2) [37]	hmmpfam is part of the HMMER suite. hmmpfam use a sequence file as it input and search against a database of hmmfiles to identify significantly similar sequence matches.	hmmpfam is used to obtain the domain architecture of a query protein sequence and its interacting partner.

8.	ScanProsite (version 1.17) [26]	ScanProsite tool allows to scan protein sequence against the PROSITE database.	Used to identify the functional sites in individual peptides. Used as a search option to identify functional patterns from the

9.	PSIPRED (version 2.5) [34]	PSIPRED is a tool for protein secondary structure from amino acid sequence based on position-specific scoring matrices	PSIPRED is used to predict the secondary structure from the sequence of interacting partner.

10	DISOPRED (version 2.1) [35]	DISOPRED is a tool for the prediction of disorder from the amino acid sequence.	DISOPRED is used to predict the disorder region from the sequence of interacting partner

11.	SEQPLOT (version 1.1) [30]	SEQPLOT is a web-utility developed to generate AAindex based plots for a protein sequence	SEQPLOT is used to generate plots using three different amino acid indices from AAINDEX database.

12.	MView (version 1.49) [43]	MView is a visualization tool for converting the results of a sequence database search into the form of a coloured multiple alignment of hits stacked against the query.	Visualization of BLASTP search results in multiple sequence alignment format is provided using MView.

### Input Details

**Figure 3:**
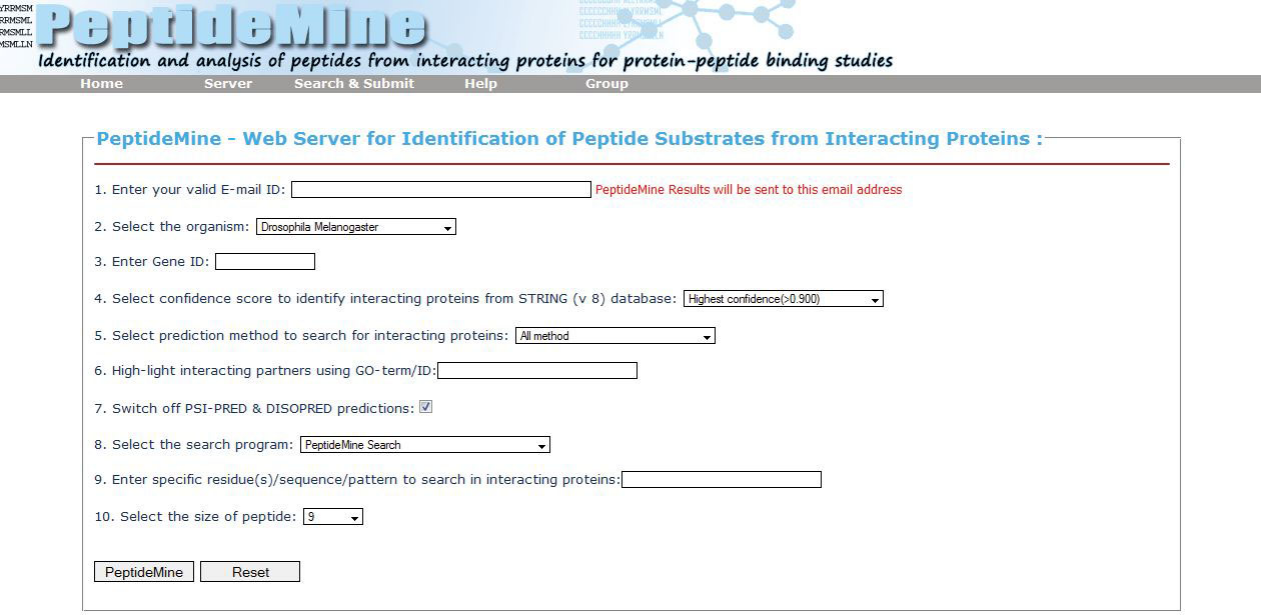
**Screenshot of the web interface of PeptideMine server**.

The input form of the PeptideMine server is designed to be highly interactive; so much so that the user can control every input parameter required for a PeptideMine search. For a successful submission of a query to the PeptideMine server, a user needs to provide the following parameters: model organism to search, Protein ID, confidence score to identify interacting proteins from the STRING database, a prediction method to search for interacting proteins and a search program. Depending upon the search program selected by the user, the related input fields will change in a dynamic way. The minimal inputs are- two for the PeptideMine Search (a residue or few residues and a specific length cut-off), BLASTP requires two input parameters (peptide sequence and E-value for BLASTP searches) and a PROSITE pattern search requires a single input of the specific pattern based on PROSITE syntax. Along with these, two optional parameters are also provided: "Highlight interacting partners using GO-term" (this option has been added for the user to provide the function or annotation of a particular protein based on a specific GO term or ID) and an optional parameter that can be used to switch-off PSIPRED and DISOPRED runs (this enables the user to obtain results very quickly). An alternate "Search and Submit" form serves as a search interface to check the back-end database whether the protein of interest is present in the current version of PeptideMine or to know about interacting partners reported in STRING database [4]. A detailed help page that explains individual parameters of the input form is provided [42]. A screenshot of the web interface of PeptideMine server is provided in Figure 3.

### Output Details

After the successful submission of input parameters, an intermediate page is generated with the details about the potential interacting partners of a query protein, GO annotations of interacting partner, number of peptides identified from individual interacting partners and a link to access the results. The GO annotations are provided for interacting partners at three levels: GO ID, GO description and GO evidence types. While the PeptideMine Search and PROSITE Search results provide links to access the results from interacting partners, BLASTP results are provided as a single link. The BLASTP search results can be visualised in a multiple sequence alignment format using MView [43].

The PeptideMine server output page is divided into three sections:

• Input parameters: Parameters used for Peptide mining from interacting proteins (Query ID, Organism, Score, Prediction Method, Residues, Search Program, Size of the Peptide, Interacting Partner, Peptides Identified, PSIPRED/DISOPRED)

• Links to download a list of peptides and Link to access hmmpfam results (predicted hmmpfam domains in the Query sequence and interacting partner). MView [43] based visualization of BLASTP result is provided along with the BLASTP result.

• Detailed output: List of Peptides Identified by PeptideMine (Number, Peptide, Start, End, Secondary Structure (PSIPRED), Disordered Region (DISOPRED), PROSITE Pattern, Instability Index, Molecular weight, pI, GRAVY, Link to SEQPLOT, Link to PMCalc, Peptide Mapped to Pfam Domains (hmmpfam), Domain-Domain interacting partner in Query Sequence (DOMINE), Link to Peptide Search in PepBank [21] and Phosphopep [44]]).

**Figure 4:**
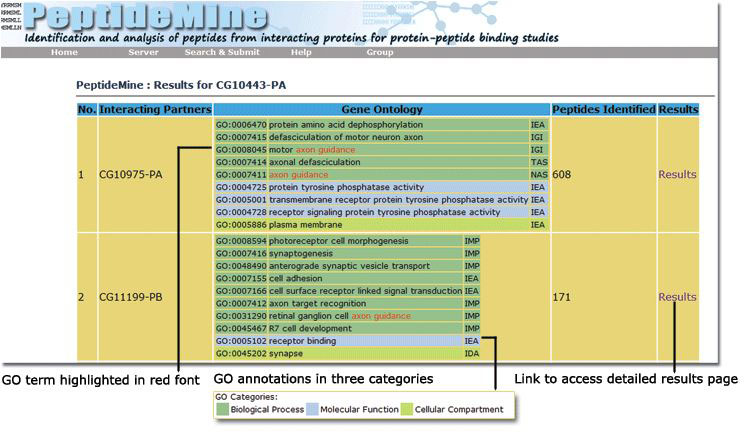
**Screenshot of a sample intermediate output page from PeptideMine server**.

**Figure 5:**
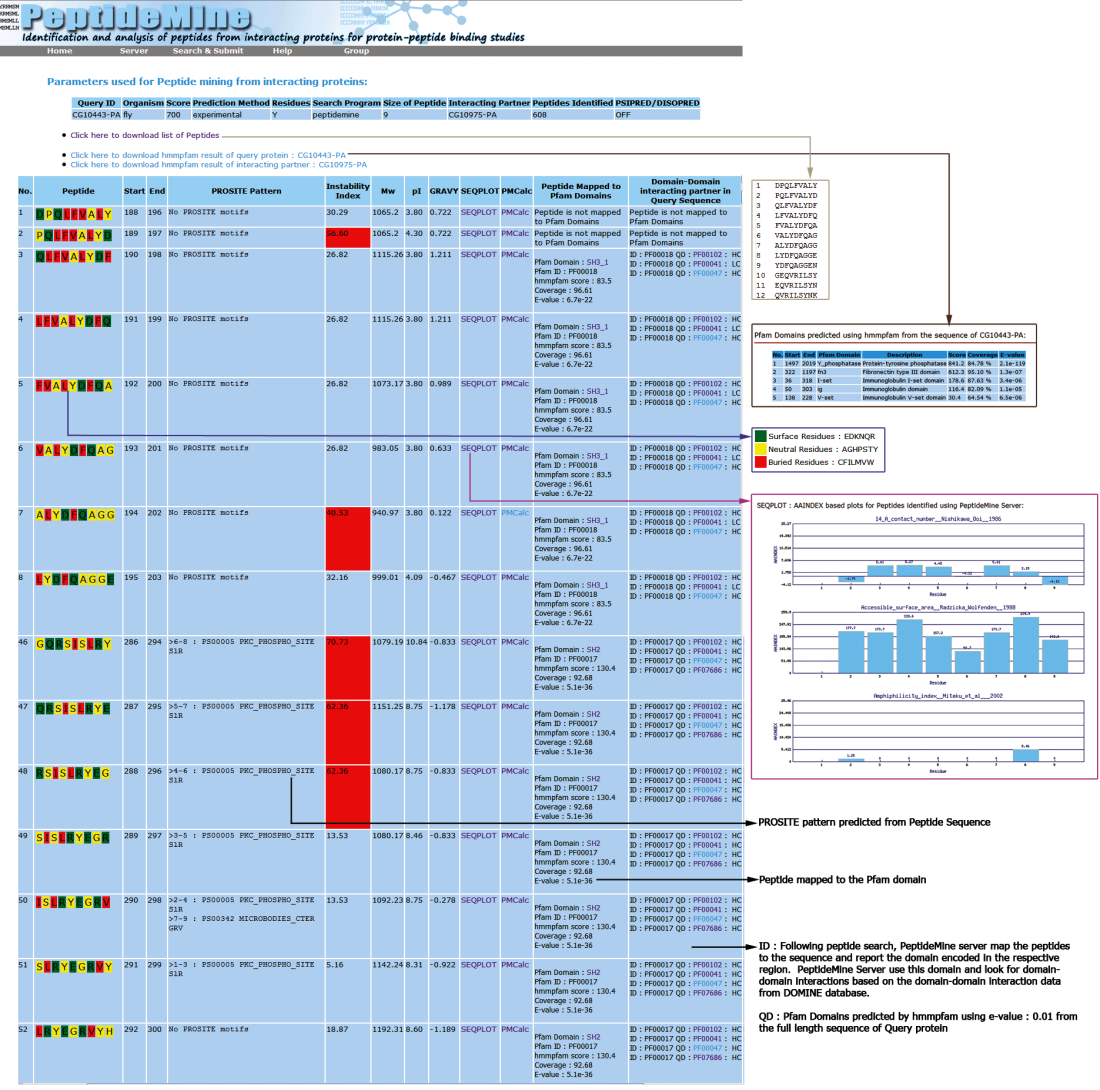
**Screenshot of a sample output page from PeptideMine server**.

The peptides presented in the output page are highlighted with three colours to reflect the hydrophobicity of the residues and the putative extent to which they are found in the interior and the exterior of proteins [45]. The classification is as follows: Surface/hydrophilic residues (Green): E, D, K, N, Q and R, Neutral residues (Yellow): A, G, H, P, S, T and Y and Buried/hydrophobic residues (Red): C, F, I, L, M, V and W. This simple visualization is likely to help the user to easily identify peptides of interest based on colour. The predicted secondary structure segments extracted from the PSIPRED output [34] and disordered region from DISOPRED [45] for the complete sequence of the interacting partner is also provided. A detailed help page that explains various features of the output is provided [46]. Screen shots of an intermediate output page that highlight the GO terms associated with interacting partners (Figure 4) and annotated screen shot of detailed output page (Figure 5) from the PeptideMine server provide a typical view of the of the results for a given query.

### PeptideMine server: technical details

The web interface (Figure 3) of PeptideMine server was developed using HTML and JavaScript. In this server, a local copy of the STRING database [4], domain-domain interaction data from DOMINE and GO data [31,32] are stored in a MySQL [47] based database. The wrapper scripts and parser for external programs like PSIPRED [34], DISOPRED [35], ScanProsite [25] AAindex [29] based graphs, 'PeptideMine Search' program, core programs for database interaction, CGI and automated e-mail notification are coded in Perl [48]. Biopython [49] based module for Protparam [50] is used to calculate peptide related parameters: instability index [27], molecular weight and theoretical pI. A dynamic charge calculator implemented using Biophp [34] is used for sequence feature calculation has been incorporated in the server. The PeptideMine server runs on an Apache webserver powered by Athlon Quad-core processors.

## Results

The PeptideMine server is designed for three main applications as mentioned earlier, *i.e*. design of peptides, search for homologous proteins with the peptide pattern and for functional motifs. In order to illustrate the use of PeptideMine in identifying polypeptide fragments for peptide libraries or sequence patterns from the interacting sequence space of a protein, we have focused on four examples. These include the receptor protein tyrosine phosphatases (RPTP), the PDZ domains from *Drosophila melanogaster*, Cyclin Dependent Kinases (CDK) and the SH3 domains from yeast. These proteins are known to play a crucial role in cell signalling and rely on protein-protein interaction for their response. However, the determinants that govern the specificity of these proteins for their interacting partners vary considerably across these examples. In the case of PTPs, the phosphotyrosine residue in the target polypeptide is often the major contributor to peptide specificity, whereas the PDZ domains have a much broader specificity determinant. Promiscuity and tolerance of different interacting partners also varies substantially across these examples; SH3 domains can bind to a large number of targets with comparable affinity. The PeptideMine results for these target proteins are detailed here. A link to access PeptideMine results of these proteins is provided in the URL [51]. The results obtained from the PeptideMine output have been experimentally validated in the case of PTP domain. These experimental data are presented along with a description of the PeptideMine output for the case of the Drosophila PTP, DLAR.

### Peptide library containing 'Tyr' residues from the interacting partners of the Drosophila Protein Tyrosine Phosphatase, DLAR

The Protein Tyrosine Phosphatases (PTP) forms the antagonistic switch of signalling mediated by the tyrosine kinases. These proteins remove a phosphate moiety from a tyrosine phosphorylated by a kinase, the identification of the substrate sequence being governed by the position of the phosphotyrosine and the nature of the flanking amino acids [52]. In this study, we examined a receptor protein tyrosine phosphatase from *Drosophila*, DLAR, and attempted to build a library of peptides from the sequence of interacting partners. The complexity of this system can be ascertained by the fact that five different RPTPs are involved in the same process of axon guidance, more so at the same stage of *Drosophila *development. The following parameters were used to perform the search in PeptideMine Gene ID: CG10443-PA (DLAR), Organism: *Drosophila*, Search Program: PeptideMine Search, PSI-PRED and DISOPRED predictions: OFF, Confidence score: 0.700, Prediction method for interaction association: Experimental, High-light interacting partners using GO-term: 'Axon guidance', Search method: "PeptideMine Search", Specific residue to search in interacting proteins: Y, Size of peptide: 10 residues. The PeptideMine server generated a list of decapeptides, each containing a Tyr residue, from within proteins involved in axon guidance. This example suggests a convenient route to design peptides for RPTPs that are of appropriate chemical parameters and are derived from biologically relevant interacting partners.

### Experimental validation of differentially charged peptides derived from the interacting sequence space of DLAR using PeptideMine server

From an experimental perspective, we have used PeptideMine server to identify differentially charged peptides from the interacting sequence space of DLAR using a combination of ScanProsite searches and BLASTP searches. Table 2 compiles the output from PeptideMine obtained using the *Drosophila *PTP DLAR as a search sequence. In an effort to determine the accuracy of the prediction as well as to obtain a characterized 'positive' identification, we included two peptides, which were already reported to be potent substrates of the RPTP DLAR (Insulin receptor peptide and Cuticle peptide), while three novel peptides were identified from the interactome of DLAR using the PeptideMine approach. The PeptideMine approach proved especially useful in finding peptides, which differed in their charge propensities around the active phosphotyrosine and hence allowed us to explore biochemical aspects of protein-peptide interaction. The activity of the recombinant DLAR catalytic domain with the five peptide substrates was ascertained by a conventional Malachite-Green assay procedure. Briefly, the inorganic phosphate released by the activity of DLAR was detected by addition of an Ammonium-Molybdate complex, which detects nmol concentrations of Pi. Table 3 provides the phosphatase activity measured by conventional malachite green assays to detect the inorganic phosphate that is released, as the peptide is de-phosphorylated. Comparable catalytic efficiencies (of the same order) for the peptides obtained from PeptideMine vis-à-vis those reported in the literature confirm that the results obtained from PeptideMine are likely to be genuine interacting partners for this protein. Various parameters and further details about the PeptideMine search results used to obtain these peptides are provided in additional file 1. URLs are provided in the Additional File 1 to access the results of the PeptideMine searches.

**Table 2:** Output from PeptideMine server with three different substrate peptides for DLAR and physiochemical properties

Peptide Sequence	Interacting partner	GO annotation	STRING Score	Instability	Molecular Weight	pI	Gravy	Mapping of peptide to a functional domain
CDDSYFGNKC	CG1560-PA myospheroid	GO:0007417 central nervous system development	0.764	57.92	1280.3	4.32	-1.145	Pfam Domain: EGF_2 Pfam ID: PF00008 hmmpfam score: 98.7 Coverage: 80.00 E-value: 8.1e-05

VIGDYVCRLCK	CG13906-PA nervous fingers 1	GO:0007411 axon guidance GO:0048663 neuron fate commitment	0.467	9.09	1269.5	8.02	0.736	Peptide is not mapped to Pfam Domains

RDDTYTAHAG	CG4032-PA Abselson tyrosine kinase	GO:0007411 axon guidance	0.232	-27.73	1177.1	5.21	-1.127	Pfam Domain: Pkinase_Tyr Pfam ID: PF07714 hmmpfam score: 530.0 Coverage: 92.65 E-value: 2.5e-156

**Table 3:** Experimental validation of the peptides derived using PeptideMine as putative substrates of DLAR

Substrate peptide	Source Protein	Search method	V_max_* (μ-mole/min/mg)	K_cat_/K_m_ (sec^-1^M^-1 ^) × 10^4^
TRDI(pY)ETDYYRK	Insulin Receptor	Literature survey	24.31 ± 0.64	26.38 ± 0.04

TAEPD(pY)GALYE	Cuticle	Literature survey	26.48 ± 1.14	30.00 ± 0.07

CDDS(pY)FGNKC	Myospheroid	PeptideMine	13.86 ± 0.39	14.28 ± 0.05

VIGD(pY)VCRLCK	Nervous Fingers	PeptideMine	12.41 ± 0.68	19.59 ± 0.11

RDDT(pY)TAHAG	Abelson	PeptideMine	3.39 ± 0.19	17.96 ± 0.16

* Phosphatase activity was measured by conventional malachite green assays to detect the inorganic phosphate that is released, as the peptide is de-phosphorylated.

### Search for canonical Cyclin Dependent protein Kinase phosphorylation motif [ST]PX[RK] in the sequence of interacting partners of YBR160W

CDK coordinates the mitogen stimulated progression of a cell from one stage in the life cycle of a cell to another. From yeast to mammals, CDKs are essential for cell cycle regulation. The interaction of these serine/threonine kinases with specific substrate proteins directly decides the fate of a cell. YBR160W is a CDC28 protein known to phosphorylate the [ST]PX[RK] motifs [53]. Cdc28 is the catalytic subunit of the main cell cycle cyclin-dependent kinase (CDK). We employed YBR160W from yeast as a query to search for the pattern [ST]PX[RK], using the PROSITE pattern search option, in PeptideMine. The server identified different instances of the pattern in 42 proteins out of the 57 interacting partners. In this example, we explain the potential use of the PROSITE pattern search in the PeptideMine server to examine functional patterns or motifs in the interacting sequence space of the CDK. Additional results from tools integrated in PeptideMine are thus likely to help the user to select the best candidate for further experimental or computational studies.

### Identification of homologous peptides from the interacting sequence space of a protein with SH3 domain

SH3 domains are important signalling domains involved in a variety of signal transduction events [54,55]. In an earlier study [56], Abp1 from yeast was shown to be a protein with a potential peptide binding SH3 domain. We used Abp1 (YCR088W) as a query with a peptide 'RPKRRAPPPVPKKP' known to interact with Abp1 and employed the PeptideMine server to search in the interacting sequence space to identify homologous peptides. A BLASTP search was performed using a relaxed e-value of 10 with the confidence score set to 0.900 and the prediction method for interaction association was selected as 'All methods'. The PeptideMine server identified 10 similar sequences from 6 out of 19 interacting partners reported in STRING. The SH3 domains are known to bind proline-rich regions [57]. We note that all the peptides obtained from the PeptideMine BLASTP search have distinct proline-rich regions. It would be interesting to experimentally evaluate these putative peptide candidates for SH3 binding, given that known sequence features that promote these interactions are conspicuously present in the target sequences. Furthermore, this list can be assessed and pruned on the basis of the physico-chemical features, if necessary, prior to experiments. This example shows an application of a BLASTP search in the PeptideMine server. Matching peptides patterns could be observed in the 19 interacting partners and the results are provided for six of them.

### Search for putative binding sites from interacting sequence space for the PDZ domain of *Drosophila melanogaster *dsh

*Drosophila melanogaster *'dsh' is known to be involved in the Wnt signalling pathway in *Drosophila*. Dsh contains a PDZ domain along with a DEP and DIX domain [58]. Proteins with PDZ domains are referred to as 'adapter' proteins and constitute one of the most commonly found protein-protein interaction domains in organisms from bacteria to humans. These proteins are crucial in signalling cascades as they form the 'links' between signalling proteins and pathways. Although they do not possess catalytic activity themselves, they are often found in conjunction with other domains that harbour kinase/phosphatases, cyclase, diesterase activity *etc*. Initially, they were expected to have a monotonous interaction of binding to the carboxy-terminal of their interaction proteins using a signature motif [FYST]-X-[FVA]. It is now known that their interaction is more diverse which includes internal protein sequences and lipids [59-61]. We used the Dsh protein from *Drosophila *and used the signature motif [FYST]-X-[FVA] to search for potential binding sites among the interacting partners of Dsh. The PeptideMine server identified binding sites among the different interacting partners. This example illustrates the combined application of PROSITE pattern search and domain-domain interaction data integrated within the PeptideMine server. Here, a known signature motif was used as a query to identify a putative binding site from the interacting sequence space. As seen by these results, peptide no. 16 mapped on to the LIM domain (Pfam ID: PF00412) and one of its corresponding interacting domain (PDZ domain, Pfam ID: PF00595) (obtained from the DOMINE database) is seen to be present in the query sequence. This could be further considered for protein-peptide docking or experimental analysis.

## Discussion

The interacting proteins identified using PeptideMine also provide a case for the examination of multi-protein associations in a cellular context. Such multi-protein complexes allow for fidelity in signal transduction events while permitting temporal association between specific proteins. In the case of proteins with multiple SH3 or PDZ domains, for example, the PeptideMine strategy can help in the prediction of the components of such cellular complexes using motif-based searches or BLASTP searches in the interacting sequence of proteins with such domains. An important caveat that we emphasize in the PeptideMine approach is that the substrate peptides are chosen 'as is'. Modification, if any, for experimentally suitable chemical properties is at the discretion of the user. This is important as naturally occurring protein-peptide interactions are tuned to their cellular function. Thus high affinity interactions are suggestive of protein association for cellular localization. Low affinity, transient interactions, on the other hand, are more suited for signal transduction events. These *in vivo *functional traits can sometimes be difficult to distinguish based on experimental strategies *in vitro *to identify target peptides. For example, in the case of a PDZ target peptide identified by phage display methods, the sequence of the selected peptide approximated the natural consensus of the *PTEN/MMAC *protein. A single residue mutation (Lys to Trp) between the natural peptide and the phage display variant led to a high affinity interaction that could be rationalized in structural terms [61]. In an earlier study, a strategy to accommodate this aspect was examined in the case of the binding affinity-selectivity model of PDZ domains based on Bayesian estimates [62]. This approach involved sequence information from both the protein and the interacting peptide. Another methodology that utilizes prior information obtained from screening random peptide libraries to filter peptide sequences that are highly unlikely to bind, has also been effectively employed in large scale proteome screening. This approach, called WISE (Whole Interactome Scanning Experiment) was reported to work well primarily due to its ability to narrow down the peptide sequence space that would be experimentally examined. Further, they have also consulted GO-term enrichment analysis for a protein and its interacting partners [63]. Our approach here is thus complementary to methodologies proposed by Chen et al. [62] and computational approach adopted by Landgraf et.al [56]. Both of these methods, described above, are not focusing on sequence searches in interacting sequence space or available as convenient webserver that can enable a user to perform such bioinformatics analysis with minimal effort. This also precludes a direct comparison of the PeptideMine server with similar tools in this specific niche. In its present form, the concept of sequence searches in interacting sequence space of proteins for peptide design is simple and effective. We anticipate that this concept, built into PeptideMine server, would be broadly applicable across different systems *albeit *with some manual intervention required to design peptides with desired characteristics.

## Conclusions

The possibility to predict and validate protein-peptide interactions is a crucial step towards identifying the links between different cellular processes. Towards this goal, the rational design of peptides that can best mimic the predicted interactions is essential to ensure experimental validation *in vitro*. PeptideMine is a generic approach to identify biologically relevant putative substrates and peptides using limited information. As the datasets and the approach are not context-biased, the PeptideMine server can be utilised effectively to identify new and potentially interesting interacting partners. This approach scores over other strategies for the identification of interacting peptides as it provides an opportunity to identify peptides from interacting partners based on user-defined residue(s) and length-based criteria along with GO annotations alongside a web-based platform for the quality assessment of peptides. Other parameters, like secondary structure, disordered regions, instability index, charge and amino acid indices, are measured for the peptides identified by PeptideMine. PeptideMine is not trained using any data that may be specific to a particular protein-peptide system. It is thus likely that the PeptideMine method and server would help identify new peptides that can be validated using experimental and computational studies potentially leading to new biological insights. The webserver will be useful for the identification of new peptides to be used in experimental and computational protein-peptide binding studies. The concept of PeptideMine and the server can be specifically used for the identification and analysis of peptides or functional patterns for binding studies, peptide design, peptide-ligand identification, identification of homologous peptides and functional motifs in the interacting sequence space of protein, compilation of peptide libraries for high-throughput screening and protein-peptide docking analysis.

## List of abbreviations used

URL: Uniform Resource Locator; GO: Gene Ontology; PTP: Protein Tyrosine Phosphatase; RPTP: Receptor Protein Tyrosine Phosphatase; CDK: Cyclin Dependent protein Kinases; SH3: Src Homology 3; dsh: dishevelled; DEP: Domain found in Dishevelled, Egl-10, and Pleckstrin; PTEN: phosphatase and tensin homolog; DLAR: *Drosophila *Leucocyte Antigen Related Protein

## Availability & Requirements

Project Name: PeptideMine - A webserver for the design of peptides for protein-peptide binding studies derived from protein-protein interactomes.

Project home page: [16]

PeptideMine results pages for examples discussed in this manuscript: [51]

Operating system(s): Platform independent webserver

License: Free for academics, Authorization license needed for commercial usage. (Please contact corresponding authors for more details)

Any restrictions to use by non-academics: license needed. (Please contact corresponding authors for more details)

## Competing interests

The authors declare that they have no competing interests.

## Authors' contributions

KS contributed to the data integration, server development, programming and compiling the manuscript, LLM, SV and BG were involved in the formulation of the strategy and representative examples, RS contributed to the overall planning of this project and the presentation of the webserver and this manuscript. All authors read and approved the final manuscript.

## Supplementary Material

Additional file 1 **Details about the PeptideMine search parameters and URL to access the PeptideMine results page of experimentally validated peptides**. Click here for file
