# Supplementary material for: PeptideMine - A webserver for the design of peptides for protein-peptide binding studies derived from protein-protein interactomes
Source: BMC Bioinformatics. 2010 Sep 22;11:473. doi: 10.1186/1471-2105-11-473 (PMC2955050; doi:10.1186/1471-2105-11-473)
Supplement: Additional file 1 — Details about the PeptideMine search parameters and URL to access the PeptideMine results page of experimentally validated peptides. [file 1471-2105-11-473-S1.DOC]

**PeptideMine – A webserver for the design of peptides for protein-peptide binding studies derived from protein-protein interactomes**

Khader Shameer^1^, Lalima L. Madan^2^, S. Veeranna^2^, B. Gopal^2*^ and Ramanathan Sowdhamini^1^*

^1^ National Centre for Biological Sciences (TIFR), GKVK Campus, Bellary Road, Bangalore, 560065, India

^2^ Molecular Biophysics Unit, Indian Institute of Science, Bangalore 560 012, India

* Corresponding authors

Email addresses: KS: [shameer@ncbs.res.in](mailto:shameer@ncbs.res.in), LLM: [lalima@mbu.iisc.ernet.in](mailto:lalima@mbu.iisc.ernet.in), SV: [veeranna@mbu.iisc.ernet.in](mailto:veeranna@mbu.iisc.ernet.in), *BG: [bgopal@mbu.iisc.ernet.in](mailto:bgopal@mbu.iisc.ernet.in), *RS: [mini@ncbs.res.in](mailto:mini@ncbs.res.in)

**Supplementary Material:**

We have used the following peptides(1-7) reported in Barr et.al [1] to identify peptides form the interacting sequence space of DLAR using PeptideMine. We included two peptides which were already reported to be potent substrates of RPTP DLAR (Insulin receptor peptide and Cuticle peptide) [2], while three novel peptides were identified from the interactome of DLAR using the PeptideMine approach. The PeptideMine approach proved especially useful in finding peptides which differed in their charge propensities around the active phosphotyrosine and hence allowed us to explore biochemical aspects of protein-peptide interaction. The activity of recombinant DLAR catalytic domain with the five peptide substrates was ascertained by a conventional Malachite-Green assay procedure. Details including the URL to access the intermediate and complete results of the PeptideMine search is provided for the seven peptides. Various parameters used for the searches is provided in the intermediate results page. Individual URLs to access the intermediate page and results pages are provided.

**1.**   IDDQYTSSGG

Both hits are from ScanProsite search using XXDYXXXXX, both hits have potential domain-domain interaction.

**Hit - 1 from PeptideMine:** CDDSYFGNKC

**Interacting Partner Details:**

**Name :** CG1560-PA / myospheroid

**GO Annotation:** Axon Guidance

**Link to FlyBase:** <http://flybase.org/reports/FBgn0004657.html>

**Link to access PeptideMine intermediate results page:** <http://caps.ncbs.res.in/peptidemine/PeptideMine_Results/peptidemine_WedApr8235621IST2009.html>

**Link to access PeptideMine complete results page:**

<http://caps.ncbs.res.in/peptidemine/PeptideMine_Results/CG1560-PAWedApr8235621IST2009.html>

**Hit -2 from PeptideMine:** **RDDTYTAHAG**

**Interacting Partner Details:**

**Name :** CG4032-PA / Abl tyrosine kinase

**GO Annotation:** Axon Guidance

**Link to FlyBase:** <http://flybase.org/reports/FBgn0000017.html>

**Link to access PeptideMine intermediate results page:**

<http://caps.ncbs.res.in/peptidemine/PeptideMine_Results/peptidemine_WedApr8235621IST2009.html>

**Link to access PeptideMine complete results page:**

<http://caps.ncbs.res.in/peptidemine/PeptideMine_Results/CG4032-PAWedApr8235621IST2009.html>

2.   AQEDFYSSRRQ

No Prosite Hits / No BLAST Hits

**3.**   HRKTYVCRMCRK

**Hit - 3 from PeptideMine:** **VIGDYVCRLCK** (Green colour portion was hit in BLASTP search with 72% similarity), we extended the peptide on left side to add 4 more residues.

Interacting Partner details:

**GO Annotation:** Axon Guidance

**Name :** CG13906-PA / nervous fingers 1

**Link to FlyBase :** <http://flybase.org/reports/FBgn0028999.html>

**Link to access PeptideMine intermediate results page:**

<http://caps.ncbs.res.in/peptidemine/PeptideMine_Results/peptidemine_ThuApr9133812IST2009.html>

**Link to access PeptideMine complete results page:**

<http://caps.ncbs.res.in/peptidemine/PeptideMine_Results/blastpeptidemine_ThuApr9133812IST2009.html>

**4.**   PKKPYDIRAD

No Prosite Hits / No BLAST Hits

**5.**   QEAYFIEDA

No Prosite Hits / No BLAST Hits

**6.**   TAEPDYGALYE

No Prosite Hits / No BLAST Hits

**7.**   TRDIYETDYYRY

No Prosite Hits / No BLAST Hits

**Reference:**

1. *Barr, A. J., Ugochukwu, E., Lee, W. H., King, O. N., Filippakopoulos, P., Alfano, I., Savitsky, P., Burgess-Brown, N. A., Müller, S., and Knapp, S. (2009). Large-scale structural analysis of the classical human protein tyrosine phosphatome. Cell, 136(2):352–363.*
2. *Cho H, Krishnaraj R, Itoh M, Kitas E, Bannwarth W, Saito H, Walsh CT. (1993) Substrate specificities of catalytic fragments of protein tyrosine phosphatases (HPTP beta, LAR, and CD45) toward phosphotyrosylpeptide substrates and thiophosphotyrosylated peptides as inhibitors. Protein Sci.2(6):977-84.*
